# Extensive rhabdomyosarcomatous differentiation in recurrent low-grade urothelial carcinoma of the bladder after transurethral resection: a case report

**DOI:** 10.1186/s13256-015-0684-7

**Published:** 2015-09-10

**Authors:** Maiko Kamei, Tsutomu Shinohara, Kotaro Kasahara, Takahira Kuno, Keishi Naruse, Hironobu Watanabe

**Affiliations:** 1 Division of Urology, National Hospital Organization National Kochi Hospital, 1-2-25 Asakuranishimachi, Kochi, 780-8077 Japan; 2 Department of Clinical Investigation, National Hospital Organization National Kochi Hospital, 1-2-25 Asakuranishimachi, Kochi, 780-8077 Japan; 3 Division of Pathology, National Hospital Organization National Kochi Hospital, 1-2-25 Asakuranishimachi, Kochi, 780-8077 Japan

**Keywords:** Sarcomatoid carcinoma, Urothelial carcinoma, Rhabdomyosarcomatous differentiation, Transurethral resection, Anaplastic lymphoma kinase

## Abstract

**Introduction:**

Sarcomatoid carcinoma of the urinary bladder is a rare bidirectional malignant neoplasm with epithelial and mesenchymal differentiation. The epithelial component is mainly high-grade urothelial carcinoma, and the mesenchymal component includes rhabdomyosarcoma. However, proper differential diagnosis of adult rhabdomyosarcomatous tumors of the bladder can be a challenge. Moreover, low-grade urothelial carcinoma as the epithelial component of sarcomatoid carcinoma has not been reported.

**Case presentation:**

A 64-year-old Asian man with a history of transurethral resection of low-grade urothelial carcinoma of the bladder visited our department with complaints of frequent urination and macroscopic hematuria. Computed tomography and magnetic resonance imaging demonstrated a large mass located in the anterior wall of the bladder. Pathological diagnosis of transurethral biopsy was low-grade, non-invasive papillary urothelial carcinoma, and tumor tissue was removed by total cystectomy. Immunohistochemical studies and fluorescence in situ hybridization assay of the resected neoplastic tissue revealed extensive rhabdomyosarcomatous differentiation causing the formation of a large pedunculated polyp with a papillary appearance of recurrent low-grade urothelial carcinoma. No evidence of recurrence was detected during 2 years of follow-up without further treatment.

**Conclusions:**

Urothelial carcinoma of the urinary bladder with extensive rhabdomyosarcomatous differentiation is rare, but it should be considered in the differential diagnosis even when urothelial carcinoma coexisting with a rhabdomyosarcomatous component is low-grade and non-invasive.

## Introduction

Sarcomatoid carcinoma (SC) is a rare bidirectional malignant neoplasm with epithelial and mesenchymal differentiation that accounts for less than 0.3% of all histological subtypes of primary urinary bladder tumors [1, 2]. The epithelial component is mainly high-grade urothelial carcinoma (UC), and the mesenchymal malignant component consists of osteosarcoma, chondrosarcoma, rhabdomyosarcoma (RMS), and so forth [1]. Inflammatory myofibroblastic tumor (IMT), also referred to as *inflammatory pseudotumor*, *pseudosarcomatous fibromyxoid tumor*, and *post-operative spindle cell nodule*, is an unusual, benign, myofibroblastic spindle cell lesion that can arise from various organs, including the urinary bladder. IMT of the bladder manifests prominent nucleoli, mitoses, chromosomal abnormalities, necrosis, and muscularis propria invasion, resembling malignant spindle cell tumors, including pure RMS, leiomyosarcoma, and SC [3, 4].

Clinically, proper differential diagnosis of adult rhabdomyosarcomatous tumors of the urinary bladder, including pure RMS, SC, and IMT, can be a challenge. In this report, we present a case of recurrent low-grade UC with extensive rhabdomyosarcomatous differentiation in an elderly man. The lesion presented as a large mass due to a rhabdomyosarcomatous component at the first medical examination. To the best of our knowledge, this is the first reported case of low-grade UC as the epithelial component of SC.

## Case presentation

**Fig. 1:**
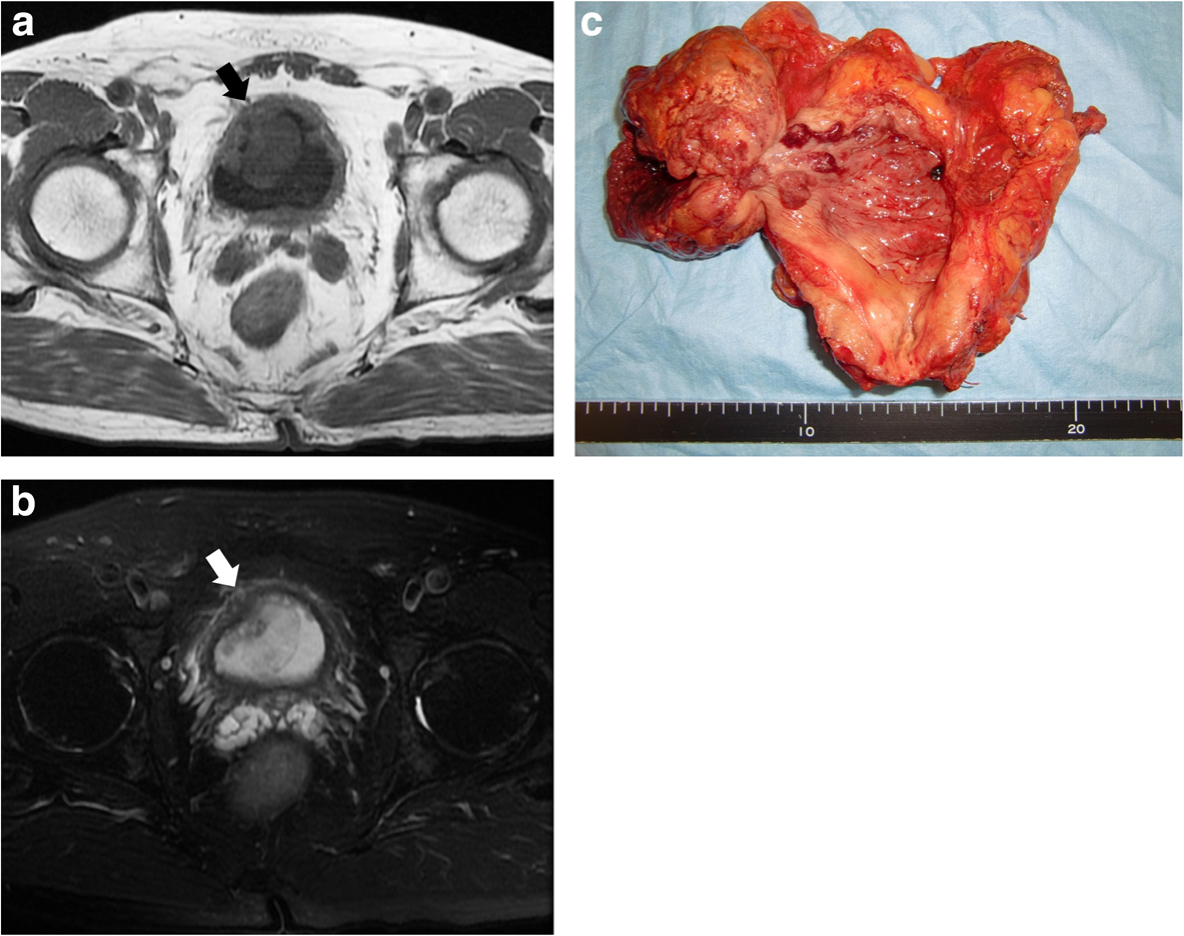
Magnetic resonance imaging scans obtained at presentation (**a** and **b**) and of the resected urinary bladder (**c**). Magnetic resonance imaging demonstrated a large mass (3.8cm×2.9cm) with fragmentation of muscularis propria (*arrows*), which was located in the anterior wall of the urinary bladder. The mass intensity was the same as in the rest of the bladder wall on T1-weighted images (**a**) and slightly greater than in the bladder wall on T2-weighted ones (**b**). The resected urinary bladder showed a large pedunculated polyp with a surrounding papillary appearance

A 52-year-old Asian man underwent transurethral resection (TUR) of stage I (T1N0M0) low-grade UC (transitional cell carcinoma [TCC]) in the anterior wall of the bladder at a nearby hospital. He was referred to our hospital 4 months after undergoing TUR. Cytoscopic examination performed at our division showed scar formation after TUR with negative biopsies, and he did not wish to undergo further treatment. Although we planned follow-up studies, he stopped visiting our hospital just after the cytoscopic examination. At the age of 64, 12 years after undergoing TUR, he visited our department with complaints of frequent urination and macroscopic hematuria without painful micturition. During his physical examination, a pelvic mass and superficial lymph nodes were not palpable, but computed tomography (CT) and magnetic resonance imaging (MRI) demonstrated a large mass (3.8cm×2.9cm) with infiltration into the muscle layer (T2b), which was located in the anterior wall of the urinary bladder. The mass intensity was the same as in the rest of the bladder wall on T1-weighted images and slightly greater than in the bladder wall on T2-weighted ones (Fig. 1a, b). Urinary cytology was positive and suggestive of UC (TCC). Cytoscopic examination revealed a large pedunculated polyp with a surrounding papillary appearance. The patient subsequently underwent a transurethral biopsy of the tumor. The pathological diagnosis was low-grade, non-invasive papillary UC (Ta), and there was no evidence of metastasis on CT or MRI. Although muscularis propria invasion was not observed in the transurethral biopsy specimens, the patient was diagnosed with stage II (T2bN0M0) UC on the basis of MRI findings of tumor infiltration into the muscle layer. Therefore, tumor tissue was removed by total cystectomy (Fig. 1c), and bilateral pelvic lymph node dissection of external iliac and obturator regions with ileal conduit urinary diversion was performed. In total, eight lymph nodes were dissected. No post-operative complications were observed.

**Fig. 2:**
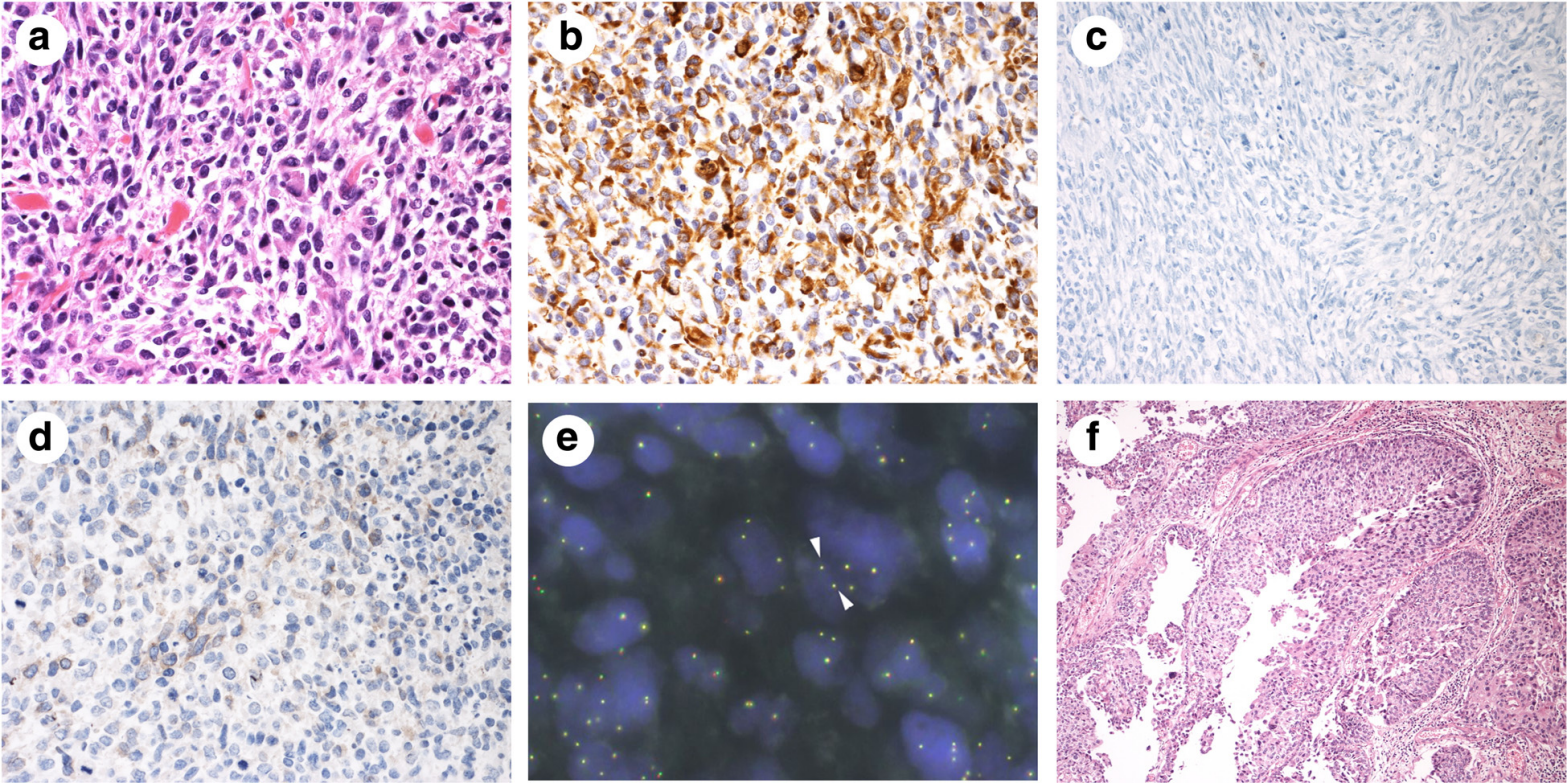
Pathological specimens of the resected neoplastic tissue. **a–e** Pedunculated polyp. **f** Papillary lesion. **a**, **f** Hematoxylin and eosin staining. **b–d** Immunohistochemical staining. **e** Fluorescence in situ hybridization using a dual-color break-apart probe for anaplastic lymphoma kinase. The 5′ end of the *ALK* gene was labeled with Vysis SpectrumGreen, and its 3′ end was labeled with Vysis SpectrumOrange (Abbott Molecular, Des Plaines, IL, USA). The pedunculated polyp consisted mainly of a sarcomatous component with a spindle cell–like appearance, which was characterized by severe nuclear pleomorphism and mitotic activity. Some strap- and racket-shaped cells with eosinophilic elongated cytoplasm were present in a dispersed manner (**a**). Spindle-like cells were positive for vimentin (**b**) but negative for cytokeratins recognized by AE1/AE3 (**c**) and anaplastic lymphoma kinase (**d**). Rearrangements and copy number gain of the *ALK* gene were not detected by fluorescence in situ hybridization. A total of 94% of tumor cells showed pseudo-color signals (*yellow color, arrowheads*) without deletion of red signals (**e**). The papillary lesion was a low-grade, non-invasive urothelial carcinoma without necrosis (**f**)

The final diagnosis of low-grade UC (TCC) with rhabdomyosarcomatous differentiation (INFa, pTa [epithelial component]/pT2b [mesenchymal component], LVI0, RM0, u-rt0, u-lt0, ur0, N0, M0) was established on the basis of immunohistochemical studies and fluorescence in situ hybridization (FISH) assay of the resected neoplastic tissue. The pedunculated polyp was made up mainly of a sarcomatous component with a spindle cell–like appearance, which was characterized by severe nuclear pleomorphism and mitotic activity (Fig. 2a), and scattered areas of necrosis. Some strap- and racket-shaped cells with eosinophilic elongated cytoplasm were present in a dispersed manner (Fig. 2a). Spindle-like cells were positive for vimentin (Fig. 2b), myogenin, and desmin, suggesting rhabdomyosarcomatous differentiation. In contrast, these cells were negative for cytokeratins recognized by AE1/AE3 (Fig. 2c), chromogranin, synaptophysin, α-smooth muscle actin, and anaplastic lymphoma kinase (ALK) (Fig. 2d). Rearrangements and copy number gain of the *ALK* gene were not detected by FISH (Fig. 2e). Muscularis propria invasion (T2b) was observed in the sarcomatous lesion without lymph node metastasis. The epithelial component of the tumor, which was consistent with a papillary lesion surrounding the pedunculated polyp, was low-grade, non-invasive UC (TCC) without necrosis (Fig. 2f). The rate of positivity of urinary cytology in patients with low-grade UC (TCC) is about 25% at our institute. The status of X chromosome inactivation and loss of heterozygosity (LOH) in both the carcinomatous and sarcomatous components was not analyzed. No evidence of recurrence was detected by whole-body CT during a 2-year follow-up period without further treatment.

## Discussion

In our patient, transurethral biopsy revealed UC but not a sarcomatous component, owing to inadequate sampling. However, the reverse situation has been reported by others. Depending on the circumstances, a small focus of carcinomatous component may be detectable only by extensive, multiple sampling. Therefore, in the case of adult rhabdomyosarcomatous tumor, the diagnosis of pure RMS should be made very carefully [5].

The other difficulty is differentiating this case from IMT. Although both epithelial and myogenic markers can be expressed in IMT, a morphologically typical epithelial component is not interwoven with IMT [4, 6]. Moreover, recent studies have shown that the majority of IMT express ALK protein, and FISH analysis has identified *ALK* gene alterations within myofibroblastic spindle cells of IMT, suggesting an etiology of a low-grade mesenchymal neoplasm other than primary inflammatory reaction [3, 4, 6]. However, our patient’s tumor was a typical epithelial carcinoma, and ALK protein expression and *ALK* alterations of the rhabdomyosarcomatous component were both negative.

The accurate histogenesis of SC has not been clarified in detail. Although multiclonal theory and monoclonal theory have been discussed, molecular and genetic research using LOH, comparative genomic hybridization, and X chromosome inactivation analysis substantiate a common origin with divergent differentiation [7]. Previous studies have suggested that the epithelial component of SC of the bladder precedes the mesenchymal component [8]. The tumor in our patient may initially have exhibited epithelial differentiation as UC and then mesenchymal differentiation with the appearance of RMS. However, it is not clear whether the origin of the tumor was residual cancer cells after TUR or de novo generation.

In most reported cases, the epithelial component of SC of the bladder is high-grade UC [9, 10]. To the best of our knowledge, low-grade UC as the epithelial component of SC has not been reported before for the present case. At times, SC occurs in patients who have undergone chemotherapy, radiotherapy, or a situation in which cell replication error was induced. However, our patient had not received any anti-cancer treatment except for TUR of low-grade UC. It has been reported that past history of non-invasive cancer correlates with the malignant grade in secondary UC [11]. In addition, the role of inflammation in urothelial cell carcinogenesis has lately been receiving considerable attention [12]. Inflammation caused by TUR for low-grade UC might have influenced the mesenchymal differentiation in UC of our patient. Indeed, others reported two cases of SC with a history of recurrent bladder cancer and subsequent TUR [10], although their report and the case of our patient do not refute the safety of TUR.

Owing to the extreme rarity of SC of the bladder and the absence of randomized controlled studies, the standard treatment for SC of the bladder has not been defined. SC usually presents at a higher T stage with more frequent regional metastases than UC [13]. Therefore, radical cystectomy seems to be preferable even in superficial disease. Although total cystectomy followed by radiotherapy or chemotherapy has been recommended by some groups [14, 15], the therapeutic efficacy of these treatments is still controversial owing to the varying results. Because muscularis propria invasion was observed in the sarcomatous lesion in our patient, we considered systemic chemotherapy (e.g., cisplatin plus gemcitabine regimen) as a treatment option. However, our patient did not wish to undergo adjuvant treatments after the total cystectomy. Nevertheless, no evidence of recurrence was detected during a 2-year follow-up period.

## Conclusions

UC of the urinary bladder with extensive rhabdomyosarcomatous differentiation is rare, but it should be considered in the differential diagnosis even when UC coexisting with a rhabdomyosarcomatous component is of low grade, especially in cases of a tumor that is unexpectedly large for low-grade UC. More molecular biological research is needed to obtain insight into the pathogenic factors involved in rhabdomyosarcomatous differentiation in UC to improve the management of this disease.

## Consent

Written informed consent was obtained from the patient for publication of this case report and accompanying images. A copy of the written consent form is available for review by the Editor-in-Chief of this journal.

## Abbreviations

ALK: Anaplastic lymphoma kinase
CT: Computed tomography
FISH: Fluorescence in situ hybridization
IMT: Inflammatory myofibroblastic tumor
LOH: Loss of heterozygosity
MRI: Magnetic resonance imaging
RMS: Rhabdomyosarcoma
SC: Sarcomatoid carcinoma
TCC: Transitional cell carcinoma
TUR: Transurethral resection
UC: Urothelial carcinoma
